# Radiographic Evaluation Factors That Influence the Decision of the Tooth Extraction Method

**DOI:** 10.7759/cureus.57746

**Published:** 2024-04-06

**Authors:** Ghassan Darwish

**Affiliations:** 1 Oral and Maxillofacial Surgery Department, Faculty of Dentistry, King Abdulaziz University, Jeddah, SAU

**Keywords:** marginal bone level, bone density, root canal anatomy, extraction of teeth, x-ray analysis

## Abstract

Introduction: A simple tooth extraction method usually involves using elevators and forceps to remove the tooth easily. In contrast, a surgical extraction method requires utilizing a straight handpiece to facilitate the tooth extraction, either removing bone or sectioning the tooth into pieces.

Objective: In this research, we aim to diagnose a tooth radiographically before extraction and determine certain factors to observe which extraction method might be more feasible, either simple or surgical.

Methodology: This study followed a retrospective cross-sectional study design. The x-ray radiographs (periapical (PA) or panoramic) were collected from the R4 system in the university dental hospital for data collection. Different radiographic influencing factors were measured, such as bone density, bone level, endodontic involvement (RCT/post and core), crowned or remaining root, and root morphology. Statistical associations were performed using SPSS (IBM Corp., Armonk, NY) with a one-way ANOVA test.

Results: There were 62 cases with 47 simple and 15 surgical extractions. There was a non-significant (p>0.05) association between the measured factors and the extraction method, either simple or surgical tooth extraction. Although bone density measurements showed a slight tendency toward PA lesions and simple tooth extraction.

Conclusion: There was no significant association between the factors and extraction methods. Future research is required to determine whether or not preoperative radiographic evaluation can influence the decision for the tooth extraction method.

## Introduction

Tooth extraction is one of the most common procedures in dental practice, which is used for the removal of a tooth from the alveolus within the alveolar bone [1]. Tooth prognosis is complex and should be established before treatment. There are different factors that cause the tooth to be extracted, systemic conditions like smoking habit, diabetes mellitus, commitment, and motivation of the patient [2]. Other local factors, such as orthodontic treatment, a periodontal compromised tooth, trauma, impaction, fracture, or non-restorable carious tooth [3]. Moreover, simple and complex extractions were performed to prevent any potential complications. Unfortunately, a lot of intact teeth have been extracted over the years [4]. Moreover, this error can be prevented by a good history and examination. Therefore, a complete diagnostic process should be discussed with the patient for any tooth planned for extraction [5].

Meanwhile, the selection of an appropriate technique for the safe extraction of the tooth without damaging any other adjacent tooth is one of the major challenges for dentists. The whole extraction procedure depends on and is influenced by the selection of technique and instrument used because the selection of the wrong instrument or inappropriate technique can convert a simple extraction into a complicated extraction [6]. Different conventional methods used for tooth extraction involve the use of luxators, elevators, forceps, and peristomes that operate on the socket expansion principle, and there are chances to traumatize the alveolar bone to some extent [7,8]. In contrast, there are other methods, such as vertical extraction systems, which have a high success rate for severely damaged tooth extraction [9,10]. Furthermore, a flap surgical method is very helpful in preserving alveolar bone, and a minimally invasive technique resulted in better preservation of the ridge [11,12]. There are other new methods, including sonic instruments for bony surgery, piezosurgery, benex extractors, endoscopically assisted root splitting, and lasers [13]. A new type of exodontia forceps uses class I lever mechanics for a tooth extraction that needs excessive force or squeezing motion for the extraction of the tooth [14]. The physical forceps can preserve the surrounding bone by minimizing root or alveolar bone fractures because they have a mechanical advantage in extracting teeth without excessive force [15]. Although previous studies have looked into the difficulty of surgery by associating it with complications related to the surgery and increased operating time, which has been the traditional method of estimating difficulty, it is currently dominated by dental factors evident in the radiographic assessment of the dentition. This is reflected in the development of three classification systems based on dental factors [16]. Assessing the complexity of a third molar surgery is essential to forming the best treatment plan to decrease complication rates. In addition, a combination of clinical and radiological interpretation is essential to reasonably estimating the time it takes to extract a tooth.

Dentists often face challenges regarding the determination of the type of treatment for affected teeth, either extraction or retention in their daily clinical practice [17]. For example, inferior alveolar nerve injury is linked with third molar surgery, so radiographic assessment is a very important initial step for the risk assessment before surgery [18]. It is also generally agreed that preoperative radiographic examination provides important information regarding roots and the surrounding tissues as well as indicates potential difficulties [19]. Moreover, radiographic examination is well justified when there is a detection or diagnosis of a condition that might change the course and mechanisms of treatment [20]. Usually, four major steps are required for a diagnosis from radiographic images: detection, recognition, discrimination, and differential diagnosis [21]. Meanwhile, a dental extraction is challenging because the clinician has to work inside the mouth, which is restricted by the patient’s cheeks, lips, and the presence of the tongue and its movement. Multiple factors can play a significant role in the complexity of the procedure, such as the dentist’s competence and practical skills and the patient’s cooperation [22].

Moreover, bone density (measured radiographically) with age shows increased surgical difficulty, which could account for the positive relationship between increased age and surgical difficulty [23]. Some anatomical, radiological, and operative variables are essential to assessing the surgical difficulty of extracting lower third molars. However, essential variables should be considered. One crucial thing that significantly influences how complicated a surgery will be is the presence of atypical root morphology and anatomy [24]. There is a high correlation between a surgeon’s expertise and postoperative complications. With careful assessment and treatment planning, postoperative complications may be minimized. Root number, morphology, tooth position, periodontal space, and second molar relationship were significant predictors of surgery difficulty [25]. All factors must be assessed, including pulpal vitality and the health of the periodontium. Sometimes, tooth extractions must be considered part of the overall treatment of the patient’s general health. For example, the treatment plan for oncology patients may involve extracting compromised teeth for those undergoing head and neck radiotherapy or chemotherapy [26].

A simple tooth extraction method usually involves using elevators and forceps to remove the tooth easily. In contrast, a surgical extraction method requires utilizing a straight handpiece to facilitate the tooth extraction, either removing bone or sectioning the tooth into pieces. This study was designed to know if it is possible to identify specific factors in the preoperative radiograph that can lead us to determine the extraction method. Although most of the research around this topic usually discusses third molar extractions. That will help both the dentist and the patient save procedure time, increase the operation’s predictability, enhance the quality of treatment, and reduce complications.

Objective

In this research, we aim to diagnose a tooth radiographically before extraction and determine certain factors to observe which extraction method might be more feasible, either simple or surgical.

## Materials and methods

Study design and setting

This study followed retrospective cross-sectional study design to investigate the radiographic evaluation factors that can influence the tooth extraction decision. This type of study approach allows data collection at a single point and offers a snapshot of these factors. Another reason was that it is very time-efficient. Moreover, this type of study also allows to measure multiple variables simultaneously and compare each of them. The study was conducted in the King Abdulaziz University dental hospital.

Sample size

A convenience sample of 62 cases comprised of 47 simple extractions and 15 surgical extractions was included in the present study. The files of the cases were sampled from Excel sheets of previous dental student cases involving fifth and sixth-year dental students.

Inclusion criteria

Healthy adults aged 18-70 years were included in the study. Specifically, I looked for cases with complete progress notes regarding how the extraction procedure was done and the extraction method.

Exclusion criteria

If it was a simple or complicated (surgical) extraction, other than that the cases was excluded from the study.

Data collection

The x-ray radiographs (periapical (PA) or panoramic) were collected from the R4 system in the King Abdulaziz University dental hospital for data collection. After collecting the desired radiographs, different important factors were interpreted, such as bone density, bone level, endodontic involvement (RCT, post, and core), crowned or remaining root, root morphology, and simple or surgical extraction.

Data collection instruments

For bone density, imageJ software was used with the grayscale unit, which is a measurement that uses the color difference to identify certain discrepancies and gives a mean value; for this method, the freehand technique was used to mark the PA area around the tooth and the surrounding bone as a reference using the same area, or Region of Interest (ROI). If there is a bone discrepancy or a lesion, it is usually the lower mean value. For bone level, this was divided into three categories: coronal third, middle third, and apical third.

Ethical considerations

The study was conducted according to the ethical principles of the Helsinki Declaration as well as ethical approval was also obtained from the university review board. Patient informed consent was obtained, and the study purpose and objectives were explained to the patients. Confidentiality and anonymity were strictly maintained throughout the study.

Data analysis

The statistical analysis was performed using a one-way ANOVA, and three different tests were used because more than one variable was to be tested, such as the chi-square test, Fisher Exact test, and independent sample t-test, to compare the different categorical and continuous variables. All statistical tests were set at the significance level of 0.05. Moreover, descriptive statistics were also performed to calculate frequencies and percentages.

## Results

**Figure 1:**
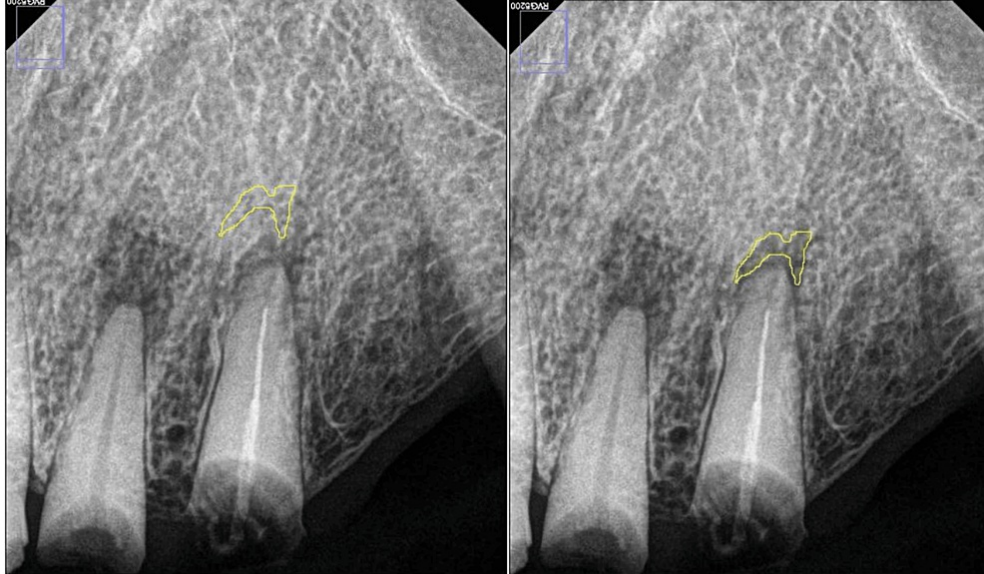
Bone density measurements using ImageJ software.

Sixty-two radiographic cases were measured accordingly for the bone level. Simple cases showed 39 (82.97%) coronal third, six (12.76%) middle third, and two (4.25%) apical third level, while surgical cases showed 14 (93.33%), 0 (0%), and one (6.66%) for coronal third, middle third, and apical third level, respectively. Statistically, there was a non-significant (p=0.33) association between bone level and method of extraction. For the endodontic treatment, simple cases had 11 (23.40%) endodontic treatments, and 36 (76.59%) had no endodontic treatment. Surgical cases had five (33.3%) endodontic treatments, and nine (60%) had no endodontic treatment. Post and core were only found in one (6.66%) surgical case, and statistically (chi-square test), a non-significant (p=0.13) association was observed between endodontic treatment and method of extraction. Furthermore, simple cases showed 38 (80.85%) remaining roots and nine (19.14%) fully crowned teeth, while surgical cases showed 12 (80%) remaining roots and three (20%) fully crowned teeth. These measurements were compared using a chi-square test, and there was a non-significant (p=0.94) association between the missing tooth crown and the method of extraction. For the root morphology, the simple and surgical cases showed 46 (97.87%) and 15 (100%) normal root morphology. Meanwhile, the resorbed root was observed in one (2.12%) simple case; for comparison, the Fisher exact test was used, and there was a non-significant association (p=0.56) between the root morphology and the extraction method. For the loss of bone density, in the case of panoramic radiographs, simple cases showed 13.8±19.4 of mean, and surgical cases showed 13.3±18.3 of mean (Figure 1), with a p-value of 0.946 indicating non-significant association. In the case of PA radiographs, simple cases showed 19.7±17.4 of mean, and surgical cases showed 9.1±22.9 with a p-value of 0.191, which showed a non-significant association between simple extractions and the PA lesion surrounding the tooth (Table 1).

**Table 1:** Association of radiographic evaluation factors with tooth extraction methods ^C^ Chi-square test ^F^ Fisher Exact test ^T^ independent sample t-test

Factors		Simple, n=47	Surgical, n=15	P-value
Bone level	Coronal	39 (82.97)	14 (93.33)	0.334 ^C^
	Middle	6 (12.76)	0 (0)	
	Apical	2 (4.25)	1 (6.66)	
Endodontic treatment	Yes	11 (23.40)	5 (33.3)	0.135 ^C^
	Yes and post	0	1 (6.66)	
	No	36 (76.59)	9 (60)	
Remaining root	Yes	38 (80.85)	12 (80)	0.942 ^C^
	No	9 (19.14)	3 (20)	
Root morphology	Normal	46 (97.87)	15 (100)	0.569 ^F^
	Resorbed	1 (2.12)	0 (0)	
Loss of bone density Panorama (mean ± SD)		13.8 ± 19.4	13.3 ± 18.3	0.946 ^T^
Loss of bone density Periapical (mean ± SD)		19.7 ± 17.4	9.1 ± 22.9	0.191 ^T^

## Discussion

The decision-making process for tooth extraction methods is a crucial aspect of daily dental practice, with radiographic evaluation playing an essential role in the establishment of effective treatment strategies [27]. Radiographs provide valuable information regarding the anatomical structures of the tooth and its surroundings. In addition, factors such as tooth morphology, proximity to vital structures, and the most important presence of any pathology are among the key considerations influencing the most appropriate tooth extraction approach [28]. Therefore, the present study aimed to diagnose the tooth based on the radiograph, looking into multiple factors that could influence the tooth extraction method. Cases can be easily identified and referred if the procedure is complicated to minimize postoperative complications and help the dentists have reliable guidelines that can be easily followed.

Based on the findings of the present study, statistically, no associations (p>0.05) were found in the studied factors. The root morphology was average in almost all analyzed cases. Thus, it was difficult to determine whether it was an essential or significant factor. In contrast, in another study, root morphology was considered a significant factor as through radiographic evaluation, a complicated root canal was confirmed [29]. Similarly, a thorough understanding of the canal and root morphology is very critical and technical before performing any procedure. Furthermore, for root canal treatment, endodontically treated teeth usually possess factors that deem the tooth more prone to fracture and thus lead to surgical extraction. However, in the present study, no influence was observed, either making the case simple or surgical. It was also observed that most of the student’s cases were remaining roots compared to full crown teeth because it is easier for students to extract and learn from them. Bone density measurements lead to some ideas. Most simple extraction cases had PA lesions compared to surgical extractions. Some surgical cases were also observed with radio-opaque or mixed lesions, which might be associated with mixed lesions or sound bone in the PA area and surgical extraction [30]. To compare the findings of the present study with other studies, there was a lack of relevant published studies; most of the studies discussed third molar extractions and the criteria regarding the position of the tooth and proximity to vital structures. However, no studies showed similar criteria or the same factors that the present study focused on. Meanwhile, other important factors were discussed in the previous studies, such as whether operator expertise correlated with postoperative complications, and they were focused on minimizing postoperative pain after dental extractions. Furthermore, how effective treatment planning can play a significant role in compromised patients. Regarding this matter, another study discussed how, unfortunately, many intact teeth had been wrongly extracted because of improper diagnosis regarding history and examination.

There are several limitations; firstly, the number of surgical cases was very low compared to the simple cases. Thus, clear associations and significance were not made in the study. Secondly, the study’s duration was approximately eight weeks from the start of the data collection until the analysis. Also, there was a lack of information in many cases found in the university dental hospital system (R4), which is why some of the important parameters were not included, and demographical information was also not clear. Many cases also need to mention the method of tooth extraction. Furthermore, more complexity and factors can be added, such as the inclusion of multi-rooted teeth, as the present study only included single-rooted teeth, and many cases available of molar teeth extracted, which were excluded.

## Conclusions

This study shed light on the significant factors that might influence the decision-making process regarding tooth extraction methods through radiographic evaluation. By analyzing different parameters like bone density, root morphology, and bone level. There was a non-significant association between these factors and the tooth extraction method. However, further research is required as the present study did not conclude whether or not it is possible to determine the extraction procedure using the radiograph only. The sample size should be increased, and both groups (simple and surgical extraction) should have an equal number of cases to have better, more accurate measurements. We also recommend using PA radiographs more than panoramic radiographs because we found out that the PA radiographs had better resolution most of the time and more predictable measurements, especially in the bone density, and to more accurately define the lesion borders as well as the surrounding healthy bone.

## References

[1] Kubilius M, Kubilius R, Gleiznys A (2012). The preservation of alveolar bone ridge during tooth extraction. Stomatol.

[2] Beck JD (1998). Risk revisited. Community Dent Oral Epidemiol.

[3] Kashif M, Mehmood K, Ayub T, Aslam M (2014). Reasons and patterns of tooth extraction in a tertiary care hospital-a cross sectional prospective survey. J Liaquat Uni Med Health Sci.

[4] Yi EKY, Ying ALS, Mohan M, Menon RK (2021). Prevalence of postoperative infection after tooth extraction: a retrospective study. Int J Dent.

[5] Dashti M, Zareh S (2021). Principles in exodontia. Innovative Perspectives in Oral Maxillofacial Surgery.

[6] Babarinde BA (2009). Selection of instruments for the successful extraction of molar teeth: a pilot study. Br J Oral Maxillofac Surg.

[7] Babbush CA (2007). A new atraumatic system for tooth removal and immediate implant restoration. Implant Dentistry.

[8] Hong B, Bulsara Y, Gorecki P, Dietrich T (2018). Minimally invasive vertical versus conventional tooth extraction: an interrupted time series study. J Am Dent Assoc.

[9] Mohamed AG, Ibrahim AE, Milad AA (2023). Tooth extraction using vertical, conventional, and surgical techniques in Sebha Dental College: a descriptive “cross-sectional study”. Open Dentistry J.

[10] Regev E, Lustmann J, Nashef R (2008). Atraumatic teeth extraction in bisphosphonate-treated patients. J Oral Maxillofac Surg.

[11] Oghli AA, Steveling H (2010). Ridge preservation following tooth extraction: a comparison between atraumatic extraction and socket seal surgery. Quintessence Int.

[12] Niyas M, Nazar N (2019). Atraumatic extractions: a revolution in exodontia-a review. Int J Clin Dentistry.

[13] Singh AK, Khanal N, Acharya N, Rokaya D, Hasan MR, Saito T (2022). Are physics forceps less traumatic than conventional forceps for tooth extraction? A systematic review and meta-analysis of randomized controlled trials. Dent J (Basel).

[14] Renton T, Smeeton N, McGurk M (2001). Factors predictive of difficulty of mandibular third molar surgery. Br Dent J.

[15] Leung YY, Hung KF, Li DT, Yeung AW (2023). Application of cone beam computed tomography in risk assessment of lower third molar surgery. Diagnostics (Basel).

[16] Jiboon AT, Alhamdani FY, Ali NH (2023). Radiographic examination before dental extraction from dentists’ perspective. Int J Dent.

[17] Dinçer B, Yetkiner E, Aras I, Attin T, Attin R (2013). Influence of lateral cephalometric radiographs on extraction decision in skeletal class I patients. Head Face Med.

[18] Alvira-González J, Figueiredo R, Valmaseda-Castellón E, Quesada-Gómez C, Gay-Escoda C (2016). Predictive factors of difficulty in lower third molar extraction: a prospective cohort study. Med Oral Patol Oral Cir Bucal.

[19] Ali K, Qazi HS, Siddiqi K, Glanville R (2021). Assessment of undergraduate students in tooth extraction competence- a cohort study. Eur J Dent Educ.

[20] Carvalho RW, do Egito Vasconcelos BC (2011). Assessment of factors associated with surgical difficulty during removal of impacted lower third molars. J Oral Maxillofac Surg.

[21] Erdelyi RA, Duma VF, Sinescu C, Dobre GM, Bradu A, Podoleanu A (2020). Dental diagnosis and treatment assessments: between X-rays radiography and optical coherence tomography. Materials (Basel).

[22] Belma Işık A, Neslihan Ün (2015). Clinical consideration and management of impacted maxillary canine teeth. Emerging Trends in Oral Health Sciences and Dentistry.

[23] Sun Y, Lu TY, Chen YC, Yang SF (2016). The best radiographic method for determining root canal morphology in mandibular first premolars: a study of Chinese descendants in Taiwan. J Dent Sci.

[24] Faraj BM, Abdulrahman MS, Faris TM (2022). Visual inspection of root patterns and radiographic estimation of its canal configurations by confirmation using sectioning method. An ex vivo study on maxillary first premolar teeth. BMC Oral Health.

[25] Udoye CI, Sede MA, Jafarzadeh H (2014). The pattern of fracture of endodontically treated teeth. Trauma Mon.

[26] Ye Z-X, Yang C, Ge J (2016). Adjacent tooth trauma in complicated mandibular third molar surgery: Rrisk degree classification and digital surgical simulation. Sci Rep.

[27] Pathak S, Vashisth S, Mishra S, Singh SP, Sharma S (2014). Grading of extraction and its relationship with post-operative pain and trismus, along with proposed grading for trismus. J Clin Diagn Res.

[28] De Bruyn L, Vranckx M, Jacobs R, Politis C (2020). A retrospective cohort study on reasons to retain third molars. Int J Oral Maxillofac Surg.

[29] Al-Haj Husain A, Stadlinger B, Winklhofer S, Bosshard FA, Schmidt V, Valdec S (2023). Imaging in third molar surgery: a clinical update. J Clin Med.

[30] Ruiz-Roca JA, Donoso-Martínez B, Ameneiros-Serantes S, Martínez-Beneyto Y, Salmerón-Martínez D, Gay-Escoda C (2020). Influence of operator's professional experience in the postoperative course after surgical extraction of the impacted lower third molar: a pilot study. J Clin Exp Dent.

